# Identification of Hepatitis E Virus Genotypes 3 and 7 in Israel: A Public Health Concern?

**DOI:** 10.3390/v13112326

**Published:** 2021-11-22

**Authors:** Rachel Shirazi, Paolo Pozzi, Yael Gozlan, Marina Wax, Yaniv Lustig, Michal Linial, Ella Mendelson, Svetlana Bardenstein, Orna Mor

**Affiliations:** 1 Central Virology Laboratory, Ministry of Health, Public Health Services, The Chaim Sheba Medical Center, Tel Hashomer, Ramat-Gan 52620, Israel; rachel.shirazi@sheba.gov.il (R.S.); yael.gozlan@sheba.gov.il (Y.G.); marina.wax@sheba.gov.il (M.W.); yaniv.lustig@sheba.gov.il (Y.L.); ellamen@sheba.health.gov.il (E.M.); 2 Department of Veterinary Sciences, University of Torino, 10095 Grugliasco, Italy; paolo.pozzi.s@gmail.com; 3 Sackler School of Medicine, Tel-Aviv University, Tel-Aviv 69978, Israel; 4 Institute of Life Sciences, The Hebrew University of Jerusalem, Jerusalem 91904, Israel; michall@mail.huji.ac.il; 5 Department of Virology, Kimron Veterinary Institute, Beit Dagan 50250, Israel; Svetab@moag.gov.il

**Keywords:** hepatitis E, HEV-3, HEV-7, swine, dromedary camel

## Abstract

Background: Hepatitis E (HEV) is an emerging cause of viral hepatitis worldwide. Swine carrying hepatitis E genotype 3 (HEV-3) are responsible for the majority of chronic viral hepatitis cases in developed countries. Recently, genotype 7 (HEV-7), isolated from a dromedary camel in the United Arab Emirates, was also associated with chronic viral hepatitis in a transplant recipient. In Israel, chronic HEV infection has not yet been reported, although HEV seroprevalence in humans is ~10%. Camels and swine are >65% seropositive. Here we report on the isolation and characterization of HEV from local camels and swine. Methods: Sera from camels (*n* = 142), feces from swine (*n* = 18) and blood from patients suspected of hepatitis E (*n* = 101) were collected during 2017–2020 and used to detect and characterize HEV sequences. Results: HEV-3 isolated from local swine and the camel-derived HEV-7 sequence were highly similar to HEV-3f and HEV-7 sequences (88.2% and 86.4%, respectively) related to viral hepatitis. The deduced amino acid sequences of both isolates were also highly conserved (>98%). Two patients were HEV-RNA positive; acute HEV-1 infection could be confirmed in one of them. Discussion: The absence of any reported HEV-3 and HEV-7 infection in humans remains puzzling, especially considering the reported seroprevalence rates, the similarity between HEV sequences related to chronic hepatitis and the HEV genotypes identified in swine and camels in Israel.

## 1. Introduction

Hepatitis E virus (HEV) is a major cause of enterically transmitted hepatitis. Of the eight genotypes of HEV, genotype 3 is the most abundantly distributed worldwide, associated with both acute and chronic hepatitis. Domestic swine and other animals are considered natural reservoirs for this genotype [1]. Genotype 7, a new genotype identified in dromedary camels, was also recently reported to cause chronic hepatitis in an immunocompromised patient [2]. In Europe, HEV is considered to be an important infection in humans, with an overall 10-fold increase in reported HEV cases over the last decade [3].

The HEV genome is a positive-sense, single-stranded RNA molecule of 7.2 kb, containing three partially overlapping open reading frames (ORF1, ORF2 and ORF3) [4]. ORF1 encodes the nonstructural proteins and enzymes, including methyltransferase, RNA helicase and RNA-dependent RNA polymerase, required for RNA replication. ORF2 and the partially overlapping ORF3 encode structural proteins. ORF2 encodes the capsid protein, which interacts with the host cell receptor. ORF3 encodes a short multifunctional phosphoprotein that can modulate cellular signaling and is related to particle secretion and considered to influence infectivity [5].

The few HEV-related cases ever to have been identified in Israel were associated with travelers returning from developing countries [6]. When molecularly assessed, HEV-1 was detected in all cases. This genotype is endemic to Asian and African developing countries, where transmission primarily occurs through contaminated water sources. Chronic infection is not associated with HEV-1. Indeed, in Israel, all the individuals with either confirmed or suspected HEV infection did not become chronically ill [6,7].

Aiming to assess a possible sub-clinical spread of the hepatitis virus in Israel, we have studied samples collected from various sewage treatment facilities (STFs) that are routinely monitored in Israel for public health purposes. Surprisingly, during 2015, HEV-3 sequences were identified, mainly in the northern region of the country, where most swine farms are located [8]. In a subsequent study, we showed that the swine are highly seropositive, the virus is endemic in local farms, and its sequence is most similar to the published HEV-3f sequences [9]. A lawsuit [10] revealed that sewage from these farms had occasionally been collected into the human sewage line, contaminating human sewage with waste from swine farms. Following the lawsuit, swine farm contamination of human wastes was resolved. Indeed, in recent years (2016–2019), analysis of sewage samples collected monthly from more than 16 STFs located through the country was always negative for the virus. As HEV-3 in swine was found to be endemic, and clinical cases were never identified in Israel, we sought to isolate the local swine derived HEV-3 virus, determine its full-length sequence and compare it to the lineages reported to cause chronic hepatitis globally.

Camels, that were recently reported to be a reservoir of a specific HEV genotype, are abundant in the southern region of Israel. In previous studies, we showed that 68.6% of the samples collected from dromedaries in Israel were anti-HEV-positive (*n* = 86 sera samples), and Bedouins who breed such camel herds had a significantly higher prevalence of HEV antibodies compared to the total population (21.6% versus 10%, respectively) [7,11]. However, the HEV genotype could not be assessed, as all the tested samples from Bedouins were HEV-RNA negative.

Here, in addition to attempting to isolate the complete HEV-3 genome from swine, we continued the search for HEV sequences using camel-derived sera. We also searched for HEV in samples from patients suspected to have HEV-related viral hepatitis. We report the nearly full-length sequences of both camel HEV-7 and swine HEV-3 and confirm the high similarity between the locally identified viruses and the published data on HEV-3 and HEV-7. We found only two cases of HEV infection in patients. Although the observations suggested that both were infected with HEV-1, we were able to confirm HEV-1 infection in only one of them. No patients with either HEV-3 or HEV-7 were identified. As HEV-3 and HEV-7 are causal agents for chronic human hepatitis disease, the apparent absence of acute or chronic infection in humans in Israel remains puzzling.

## 2. Materials and Methods

### 2.1. Samples Used for Identification of HEV

Remains of blood (*n* = 3) and feces (*n* = 18) samples from 2.5–4-month-old swine, collected during 2015–2016 to study the endemicity of HEV in local farms and found to be HEV-positive [9], were used here for full-length sequencing of the swine-derived viral genome.

Sera samples collected in 2018 from 142 local dromedary camels (as part of the Middle East Respiratory Syndrome, MERS, National Control Programme), were used for HEV analysis. Serological screening was performed prior to molecular analysis using an HEV-Ab ELISA kit (Wantai, Biologic Pharmacy Enterprise, Beijing, China), which measures total antibodies and is suitable for detecting anti-HEV antibodies in non-human sera. 

Total RNA was extracted from 400 μL camel sera and assessed with the RealStar RT-PCR HEV kit 2.0 (Altona Diagnostics GmbH, Hamburg, Germany), for which the lowest limit of detection is 100 IU/mL. The kit is capable of identifying all relevant HEV genotypes.

### 2.2. Screening for HEV among Human Patients

Blood samples from patients suspected to have HEV-related viral hepatitis were transferred to the National HIV and viral hepatitis Reference Laboratory (NHRL) for confirmation of HEV infection. Total RNA was extracted and assessed using the RealStar HEV kit as described above. RNA from positive samples was further analyzed by nested RT-PCR and sequenced to determine the HEV genotype, using the universal nested RT-PCR assay capable of detecting divergent strains of HEV [12].

### 2.3. Characterization of HEV-3 and HEV-7 Genome by Population Sequencing

Published primer sequences spanning the whole HEV-3 and HEV-7 genome (Supplementary Table S1) were used for amplification and population sequencing of the HEV genomes. Both strands of all PCR amplicons were sequenced to minimize sequencing errors. All sequence chromatograms were assembled and edited manually, using GeneObject software (Visible Genetics Inc., Toronto, ON, Canada).

### 2.4. Phylogenetic Analysis of Camel and Swine-Derived HEV

For phylogenetic analysis the sequences of camel- and swine-derived HEV and full-length reference HEV sequences retrieved from GenBank were aligned using Clustal X and manually modified as required. Using Mega 7.0 [13], an unrooted maximum likelihood tree was inferred by the neighbor-joining method. Bootstrap analysis was performed with 1000 replicates. The local HEV sequences were also assessed by the RIVM HEV genotyping tool (https://www.rivm.nl/mpf/typingtool/hev/introduction, accessed on 22 August 2021).

### 2.5. Sequence Comparison

Camel HEV-7 sequences and a total of nine full-length HEV-3 sequences, representative of the most similar genotype 3 subtypes, were retrieved from GenBank and compared to local HEV-3 and HEV-7 sequences. Amino acid sequences were deduced from aligned nucleic acid sequences using SEQUENCHER 5.4 (GeneCodes, Ann Arbor, MI, USA). Nucleotide sequences from HEV-3f and HEV-7 isolates described in this study were submitted to GenBank (MZ983634-MZ983635).

## 3. Results

### 3.1. Identification of HEV-7 in Camel Sera

In an attempt to determine whether local dromedaries are infected with HEV, remains of camel sera collected in 2018 (*n* = 142) were screened for anti-HEV IgG antibodies and for HEV RNA. While 54% (77/142) were IgG-positive, only a single HEV seropositive sample was also HEV-RNA-positive (Supplementary Table S2). Initial phylogenetic analysis based on a 2508 nucleic acid-long gene fragment amplified from the 3′ segment of the genome (positions 4578–7085 bp), revealed that this sequence clustered with camelid HEV-7 sequences KJ496143 and KJ496144, and also with a HEV sequence from a patient with viral hepatitis (KT818608), all retrieved from GenBank.

### 3.2. Analysis of Camel- and Swine-Derived HEV Sequences

**Figure 1:**
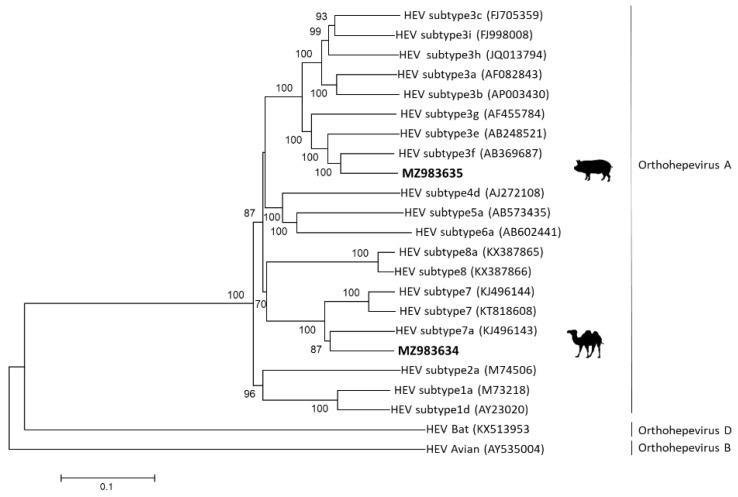
Phylogenetic tree of local HEV from camel and from swine. The tree was constructed using the maximum likelihood method with MEGA7. Bootstrap values of >70% are presented. The analysis included the whole genomes of GenBank HEV reference sequences (HEV-1-8), shown in parentheses. The GeneBank assignment for local camel HEV-7 is MZ983634 and for local swine HEV-3f is MZ983635.

The nearly full-length camel-derived HEV genome was successfully sequenced from the sample that had a high enough viral load (cycle threshold, Ct = 27.3). The whole genome of swine-derived HEV was successfully sequenced from a fecal sample (Ct = 24.9). Previously, using partial sequencing, we have shown that swine-derived HEV clustered with HEV-3f sequences [9]. Figure 1 presents the results of the phylogenetic analysis of the nearly whole genome of the local camel- and swine-derived HEV sequences isolated here.

Next, the genome properties of the HEV-3f and HEV-7 viruses were compared with similar HEV sequences (Table 1). The overall sequence similarity of local HEV-3 and HEV-7 genomes with the matched reported isolates was high. The similarity between the three open reading frames of both viruses to related family members was also high. The similarity with the local HEV-3f ranged between 81.4% and 99.2% for ORF1, 98.8–99.6% for ORF2 and 96.7–98.9% for ORF3. For the local HEV-7, it ranged between 81.3% and 81.4% for ORF1, 99.3–99.4% for ORF2 and 98.2–99.4% for ORF3.

Domain analysis (Table 2) revealed that although the nucleic acid and putative protein sequence of ORF1 was the most diverse compared to the other ORFs, all functional domains were conserved in both HEV-3f and in HEV-7 local sequences. Swine ORF2 (660 amino acids) had three unique amino acid substitutions: G23S, P25S and A72T. The A606V substitution was identified in another HEV-3f isolate (AOG18225). H99L, I102T and S110P, the three positions that were mutated in ORF3, alter the polarity of these amino acids; however, none of these sites was previously associated with any specific protein function. The predicted 3D structural models of the local HEV-3f ORF2 and ORF3 were identical to the models based on the closely-related published isolates.

The local predicted HEV-7 ORF2 capsid protein had few substitutions. Most of the substitutions were located in the N′-terminal region and included non-conservative changes, e.g., S35G, S68A and P70S, all located within the 111-amino acid N terminal region shown to be responsible for the inhibition of interferon production and thus considered to be important for evasion of the host innate immune system [14]. A conservative change (I113V), as well as a single substitution (S149A) in the shell domain (spanning amino acids 129–319), were also observed.

Local HEV-7 ORF3 exhibited a single conservative substitution, V92A, in a site located between the two conserved PSAP motifs (amino acids 86–89 and amino acids 95–98). Mutations in these motifs were previously associated with a decrease in HEV replication [5]. Aside from these few amino acid mutations in both ORF2 and ORF3, all other residues which were previously shown to be of relevance to HEV infection, replication and expression of the capsid protein were conserved.

### 3.3. Analysis of Serum Samples from Patients with Acute Viral Hepatitis

Serum samples collected between 2018 and 2020 from patients suspected to have viral hepatitis were tested for the presence of HEV RNA. Overall, of the 101 samples tested (52, 29 and 20 samples in 2018, 2019 and 2020, respectively), only two samples were HEV-positive. One was a 23-year-old returning male traveler arriving from India, proven to be infected with HEV-1. The other was a local 64-year-old man diagnosed with acute viral hepatitis and low levels of HEV-RNA (Ct = 36.2), which was not sufficient for sequencing. Neither HEV case became chronic.

## 4. Discussion

In this study, we isolated HEV-3 and HEV-7 sequences from swine and from dromedaries, respectively, and showed that the nearly full-length nucleotide sequences of local strains were highly similar to publicly available HEV-3 and HEV-7 sequences. Previously, analysis of nucleotide *p*-distances of all available complete HEV genome sequences and the assignment of reference sequences for each subtype enabled the classification of HEV-3 into 10 subtypes [15]. Using a fragment of the swine-derived HEV, we have already shown that it branches with the HEV-3f subtype [9]. Here, we corroborated this finding, and using the full-length nucleic acid sequence of the local swine HEV-3, showed that it was indeed most similar (88.2% identity) to HEV-3f. The overall nucleotide sequence of the local HEV-7 sequence was also similar (86.4% identity) to a published HEV-7 sequence from a camel and the phylogenetic analysis showed that it branched with the other reported HEV-7 sequences.

Overall, the deduced amino acid sequences of the three ORFs of both local genotypes were almost identical to the ones identified in other viral isolates from other countries (99%). ORF2 and ORF3 were more conserved than ORF1. Indeed, conservation of ORF3 is to be expected, as this protein was recently demonstrated to be an ion channel involved in the release of infectious virions [16]. Similar predictions were made for the transmembrane domain in swine and camel ORF3 proteins. Moreover, the PSAP functional motif, which is involved in virion release by allowing it to interact with endosomal sorting complexes (ESCRT), is conserved [17]. The few amino acid changes between both HEV species were insignificant and did not alter the overall predicted 3D protein structures observed in other reported HEV-3 and HEV-7 isolates.

The sequence and structural conservation of all three ORFs suggests that the function of the deduced proteins is maintained. Heterogeneity of protein domains and sequence variability is expected to significantly affect the pathogenesis and severity of infection. It was also suggested that genetic heterogeneity enables the virus to better adapt to the host and persist longer, and thus establish chronicity [18]. Indeed, in silico analyses have attempted to establish a link between the HEV sequence and viral host preference or transmissibility [19,20]. However, functional confirmation of any such hypothesis is still pending and is currently difficult to pursue. The virus is difficult to culture from clinical specimens (we failed to culture the swine and the camel-derived viruses), and animal models are very limited; therefore, sequence analysis remains a key approach for prediction of possible functions.

Considering the seroprevalence of 10% in the Israeli population, asymptomatic HEV-3 or HEV-7 infections cannot be ruled-out. Misreporting is also possible, although, with the publicity of chronic hepatitis HEV-3 cases in Europe [21,22,23,24,25,26,27], local health authorities’ awareness of this type of viral infection is high. The role of different food consumption patterns between Israeli inhabitants with different religious backgrounds and their risk of being seropositive should also be further studied.

It was already suggested that a One Health approach is necessary to understand and mitigate HEV transmission. Swine and camels infected with HEV display no obvious clinical symptoms and identification of HEV in such animals does not require any disease-control measures. However, both species shed the virus into the environment in feces, from which it can be transferred to another host. Moreover, products from both swine and camels are consumed by humans.

Indeed, the European Centre for Disease Prevention and Control (ECDC) and the European Food Safety Authority (EFSA) identified a need to support activities related to the investigation and assessment of HEV in the EU/EEA [3,28]. Our study showed that although no clinical cases of HEV-3 or HEV-7 infection were reported, these viruses do exist in Israel. Therefore, future molecular and epidemiological efforts aiming to detect HEV infection should be continued. Since HEV-3 has been transmitted between humans through blood transfusion, testing of blood donors in European countries has been introduced [29,30]. In Israel, HEV-7 is yet another public health risk, especially for those who are immunocompromised. Screening for HEV-3 or HEV-7 in all blood donations in Israel should also be considered.

## Figures and Tables

**Table 1:** (a) Comparison of the nucleotide and deduced amino acid sequences of local swine HEV-3 versus reported swine HEV-3 subtypes. nt—nucleic acids; aa—amino acids. (b) Comparison of the nucleotide and deduced amino acid sequence identities of local camel-derived HEV-7 versus reported camel HEV-7.

**(a)**							
***HEV Swine***							
**Genotypes/Strains** **(GenBank Accession)**	**Nucleotide Identity (%)**				**Amino Acid Identity (%)**		
	**Whole Genome**	**ORF1** **(5112 nt)**	**ORF2** **(1980 nt)**	**ORF3** **(366 nt)**	**ORF1** **(1704 aa)**	**ORF2** **(660 aa)**	**ORF3** **(122 aa)**
HEV subtype 3a (AF082843)	81.4	79.9	84.8	92.1	81.7	98.8	96.7
HEV subtype 3b (APOO3430)	80.9	79.4	84.4	92.7	81.8	98.8	97.6
HEV subtype 3c (FJ705359)	81.0	79.2	85.2	94.3	81.9	99.2	98.1
HEV subtype 3e (AB248521)	84.7	83.3	88.2	95.7	82.4	99.4	98.4
HEV subtype 3f (AB369687)	88.2	87.3	90.4	96.7	99.2	99.5	98.9
HEV subtype 3f (MH504151)	87.5	87.1	88.5	96.2	82.3	99.6	98.6
HEV subtype 3g (AF455784)	77.8	74.6	85.7	93.2	81.4	99.2	98.4
HEV subtype 3 (KY232312)	87.3	86.5	89.0	94.3	99.2	99.2	98.9
HEV subtype 3 (MH450029)	87.4	86.5	89.4	96.2	99.1	99.4	98.9
**(b)**							
***HEV Camel***							
**Genotypes/Strains** **(GenBank Accession)**	**Nucleotide Identity (%)**				**Amino Acid Identity (%)**		
	**Whole Genome**	**ORF1** **(5046 nt)**	**ORF2** **(1980 nt)**	**ORF3** **(339 nt)**	**ORF1** **(1682 aa)**	**ORF2** **(660 aa)**	**ORF3** **(113 aa)**
HEV subtype 7 (KJ496144)	85.0	83.9	87.7	94.7	81.3	99.3	98.2
HEV subtype 7a (KJ496143)	86.4	85.4	88.8	95.6	81.4	99.4	99.4

**Table 2:** Differences in deduced amino acids between local and published sequences.

**a. Differences in HEV-3 Subtypes at ORF2 and ORF3**								
**HEV-3 Subtypes**	**ORF2, Amino Acid**				**HEV-3 Subtypes**	**ORF3, Amino Acid**		
	**23**	**25**	**72**	**606**		**99**	**102**	**110**
HEV_Swine_ISRAEL	**S**	**S**	**T**	**V**	HEV_Swine_ISRAEL	**H**	**I**	**S**
HEV_AAC97210_3a	G	P	A	A	HEV_AAC97209_3a	L	T	P
HEV_BAB63941_3b	G	P	A	A	HEV_BAB63940_3b	L	T	P
HEV_ACR56298_3c	G	P	A	A	HEV_ACR56299_3c	L	**I**	**S**
HEV_BAE98089_3e	G	P	A	A	HEV_BAE98088_3e	L	T	P
HEV_ACU12605_3f	G	P	A	A	HEV_QBG59341_3f	L	T	P
HEV_AOG18222_3f	G	P	A	A	HEV_AOG18224_3f	**H**	T	P
HEV_AFH35001_3f	G	P	A	A	HEV_AFH35000_3f	L	T	P
HEV_BBB44460_3f	G	P	A	A	HEV_AFH35003_3f	L	T	P
HEV_BAT32887_3f	G	P	V	A	HEV_BBB44459_3f	L	T	P
HEV_AOG18225_3f	G	P	A	**V**	HEV_BAT32886_3f	L	T	P
HEV_QBG59342_3f	G	P	A	A	HEV_AOG18221_3f	L	T	P
HEV_BAG32126_3f	G	P	A	A	HEV_ACU12606_3f	L	T	P
HEV_AFH35004_3f	G	P	A	A	HEV_AAO67360_3g	L	T	P
HEV_AAO67359_3g	G	P	A	A	HEV_AEX65898_3h	L	T	P
HEV_AEX65899_3h	G	P	A	A	HEV_ACV66471_3i	L	T	P
HEV_ACV66470_3i	G	P	A	A				
**b. Differences between HEV-7 Subtypes at ORF2 and ORF3**								
**HEV-7 Subtypes**	**ORF2, Amino Acid**						**ORF3, Amino Acid**	
	**35**	**68**	**70**		**113**	**149**	**92**	**108**
HEV_Camel_ISRAEL	**S**	**S**	**P**		**I**	**A**	**A**	L
HEV_AHY61299_7	G	A	S		V	S	V	L
HEV_AHY61296_7a	G	A	S		V	S	V	H

## Data Availability

All data supporting reported results can be provided upon request.

## Supplementary Materials

The following are available online at https://www.mdpi.com/article/10.3390/v13112326/s1, Table S1: Primers used to amplify hepatitis E viral genome of genotypes 3 and 7, Table S2: IgG and HEV-RNA results in serum from dromedary camels (*n* = 142).
