# Supplementary material for: Identification of Hepatitis E Virus Genotypes 3 and 7 in Israel: A Public Health Concern?
Source: Viruses. 2021 Nov 22;13(11):2326. doi: 10.3390/v13112326 (PMC8625709; doi:10.3390/v13112326)
Supplement: Supplementary file 1 [file viruses-13-02326-s001.zip › viruses-1477874-supplementary.pdf]

Supplementary materials

Table S1. Primers used to amplify hepatitis E viral genome of genotype 3 and 7.

Virus Targeted	Sequence (5‘-3’)	Position	Polarity	Reference
HEV-3	ATGTARTCACGGCCDGACTTCTC	1302–1279	antisense	*Bo Wang et al. (2018)*[1] and unpublished primers received from Bo Wang
HEV-3, HEV-7	TCTAATGGCCTGGACTGTACTG	1894–1915	sense	
HEV-3	ACYTGGTCHACATCTGGYTTYTC	2139–2161	sense	
HEV-3	TAYCCTGATGGRGCYAAGGTGT	2995–2973	sense	
HEV-3	TTAACCARCCARTCACARTCYGAYTCAAA	3037–3009	antisense	
HEV-3	TGRACCGATGAGGCKCGCTGCAT	3200-3222	antisense	
HEV-3, HEV-7	TACCACCAGCTKGCTGAGGAG	3751-3771	sense	
HEV-3	ATGGAGGAGTGTGGCATGC	4465-4483	sense	
HEV-3, HEV-7	GCCATGTTCCAGACDGTRTTCCA	4622-4600	antisense	
HEV-3	GAAGGGGTTGGTTGGATG	5332-5315	antisense	
HEV-3	TTCATCCAACCAACCCCT	5367-5383	sense	*Garson et al. (2012)*[2]
HEV-3	AATTATGCYCAGTAYCGRGTTG	5783-5804	sense	*Huang et al. (2002)*[3]
HEV-3	GTYATGCTYTGCATWCATGGCT	6068-6089	sense	
HEV-3	AGCCGACGAAATCAATTCTGTC	6394-6415	antisense	
HEV-3	CCCTTRTCYTGCTGWGCATTCTC	6491-6513	antisense	
HEV-7	AYAACCATGATGTTGCCATACT	742-763	sense	*Lee GH et al. (2016)*[4]
HEV-7	GTAAAACGACGGCCAGGGTAGACCACGTACGTTGCT	2103-2122	antisense	
HEV-7	TCGCCGAATCATATCTGGGA	2103-2122	sense	
HEV-7	AGGGGATGAGCTTTATCTSA	2798-2817	sense	
HEV-7	TSAGATAAAGCTCATCCCCT	2798-2817	antisense	
HEV-7	TACWATCATTGCCACAGCTGA	3527-3547	sense	
HEV-7	TCAGCTGTGGCAATGATWGTA	3527-3547	antisense	
HEV-7	TATATTCATCCAACCAACCCAT	5334-5375	sense	
HEV-7	ATGGGTTGGTTGGATGAATATA	5334-5375	antisense	
HEV-7	TTGATATGAATTCAATYACTTCGA	5856-5879	sense	
HEV-7	TCGAAGTRATTGAATTCATATCAA	5856-5879	antisense	
HEV-7	ACGAAATCAATTCTGTCGGGA	6339-6359	antisense	
HEV-7	CAGGAAACAGCTATGACTTTTCAGGGAGCGCGAAACG	7226-7262	antisense	
HEV-3, HEV-7	TAGGCAGACCACRTATGTGGTCGSTGCCATGGA	1-990	sense	*Legrand-Abravanel F el al. (2009)*[5]
HEV-3, HEV-7	GCCGGTCCCAGATRTGSACCGGRA	1-990	antisense	
HEV-3	ACGAATGTYGCGCAGGTYTGTGT	5003-6484	sense	

HEV-7	TTTATTCTCGTCCAGTCGTTTC	6358-6379	sense	*P.C.Y Woo et al (2014)*[6]
HEV-7	GTCAGTGGAGGACCCATATGT	6620-6641	antisense	
HEV-3, HEV-7	CTGGCATYACWACTGCYATTGAGC	56-79	sense	*Schlauder et al. (1999)*[7]
HEV-3, HEV-7	CCATCRARRCAGTAAGTGCGGTC	473-451	antisense	
HEV-3	TCTACATTTCATGCTGTWCCGGT	958-980	sense	*This study**
HEV-3, HEV-7	CTGCCATCAGCGCTACCTNCGWAC	1170-1193	sense	
HEV-3	TGTGGYCAYGATAACGAGGCCT	1513-1534	sense	
HEV-3	AGGCCTCGTTATCRTGRCCACA	1513-1534	antisense	
HEV-3, HEV-7	GGGTTGAGCAGAACCCYAAGAGG	2696-2718	sense	
HEV-3, HEV-7	CGATGGTTACGCTCCCAGGC	2774-2793	antisense	
HEV-3	TACTGCGTGAGGTCGGCATT	3260-3659	sense	
HEV-3	CCTTCTCMACCATAGCCTC	4132-4150	antisense	
HEV-3	GCTTGAAGAYACYATTGA	7022-7039	sense	
HEV-3	TAATTAAGACTCCCGGGT	7164-7181	antisense	
HEV-7	CTAGCTGGTGGCACGTTACCC	3290-3310	sense	
HEV-7	CATGARGCGCAGGGRGCGAC	3479-3494	sense	
HEV-7	CAATGGACTATATCAGTGAGC	3915-3935	antisense	
HEV-7	CTAGTTGGCAGATATGGC	3979-3996	sense	
HEV-7	ATAGCGCATTGTTATGAGTTCC	4654-4675	sense	
HEV-7	CAAGACGAAGCTGTTCAGC	4915-4933	antisense	
HEV-7	CTTGGCGYGACCAATCCC	5428-5445	sense	
HEV-7	ACCAGTATAGGGTGTATTA	6038-6056	antisense	

* Primer positions in this study are based on GenBank accession numbers AB369687 for HEV-3 sequences and KJ496143 for HEV-7 sequences

Table S2. IgG and HEV-RNA results in serum from dormitory camels

Tribe/Location	Collection year	Gender (F/M)	Estimated age (Year)	Number of sera from camels	IgG Positive (n)	RNA Positive (n)
Mas'udein el-Azazme	2018	12F; 1M	3	13	7	0
		F	3	10	9	0
		F	3	4	0	0
		F	3	3	0	0
		6F;1M	3	7	4	0
Abu Rakeek	2018	F	3	5	0	0
		F	3	5	1	0
Kfar Adumim	2018	10F; 1M	Unknown	11	8	0
Atrash	2018	5F; 1M	6	5	2	0
Mas'udein el-Azazme	2018	22F; 1M	1	23	18	1 (*Ct= 27.4*)
Segev Shalom	2018	F	6	3	0	0
		F		3	0	1
Tel Sheva	2018	3F; 1M	6	4	2	0
		F	3	2	0	0
Yeruham	2018	F	3	9	8	0
		F	3	3	3	0
Arad	2018	F	6	0	0	0
		F	6	1	0	0
		F	6	2	0	0
Hawashla	2018	F	6	4	3	0
Ze'elim	2018	F	3	1	0	0
Uum batin	2018	F	3	7	5	0
Ar'ara	2018	5F; 1M	6	5	3	0
Unknown	2018	F	6	3	0	0
Abu Rbei'a	2018	F	3	3	2	0
Kseifa	2018	F	3	6	2	0

References:

1. Wang B, Harms D, Papp CP, Niendorf S, Jacobsen S, Lütgehetmann M, Pischke S, Wedermeyer H, Hofmann J, Bock CT. **Comprehensive Molecular Approach for Characterization of Hepatitis E Virus Genotype 3 Variants.** J Clin Microbiol. 2018 Apr 25;56(5):e01686-17. doi: 10.1128/JCM.01686-17. PMID: 29514938; PMCID: PMC5925713.

2. Garson JA, Ferns RB, Grant PR, Ijaz S, Nastouli E, Szypulska R, Tedder RS. **Minor groove binder modification of widely used TaqMan probe for hepatitis E virus reduces risk of false negative real-time PCR results.** J Virol Methods. 2012 Dec;186(1-2):157-60. doi: 10.1016/j.jviromet.2012.07.027. Epub 2012 Jul 31. Erratum in: J Virol Methods. 2014 Dec;209:143. PMID: 22871672.

3. Huang FF, Haqshenas G, Guenette DK, Halbur PG, Schommer SK, Pierson FW, Toth TE, Meng XJ. **Detection by reverse transcription-PCR and genetic characterization of field isolates of swine hepatitis E virus from pigs in different geographic regions of the United States**. J Clin Microbiol. 2002 Apr;40(4):1326-32. doi: 10.1128/JCM.40.4.1326-1332.2002. PMID: 11923352; PMCID: PMC140370. Detection by Reverse Transcription-PCR and Genetic Characterization of Field Isolates of Swine Hepatitis E Virus from Pigs in Different Geographic Regions of the United States

4. Lee GH, Tan BH, Teo EC, Lim SG, Dan YY, Wee A, Aw PP, Zhu Y, Hibberd ML, Tan CK, Purdy MA, Teo CG. **Chronic Infection with Camelid Hepatitis E Virus in a Liver Transplant Recipient Who Regularly Consumes Camel Meat and Milk. Gastroenterology**. 2016 Feb; 150(2):355-7.e3. doi: 10.1053/j.gastro.2015.10.048. Epub 2015 Nov 6. PMID: 26551551.

5. Legrand-Abravanel F, Mansuy JM, Dubois M, Kamar N, Peron JM, Rostaing L, Izopet J. **Hepatitis E virus genotype 3 diversity, France**. Emerg Infect Dis. 2009 Jan; 15(1):110-4. doi: 10.3201/eid1501.080296. PMID: 19116067; PMCID: PMC2660688.

6. Woo PC, Lau SK, Teng JL, Tsang AK, Joseph M, Wong EY, Tang Y, Sivakumar S, Xie J, Bai R, Wernery R, Wernery U, Yuen KY. **New hepatitis E virus genotype in camels, the Middle East.** Emerg Infect Dis. 2014 Jun; 20(6):1044-8. doi: 10.3201/eid2006.140140. PMID: 24856611; PMCID: PMC4036782.

7. Schlauder GG, Desai SM, Zanetti AR, Tassopoulos NC, Mushahwar IK. **Novel hepatitis E virus (HEV) isolates from Europe: evidence for additional genotypes of HEV.** J Med Virol. 1999 Mar; 57(3):243-51. doi: 10.1002/(sici)1096-9071(199903)57:3<243::aid-jmv6>3.0.co;2-r. PMID: 10022795.
